# Medically Defined Infertility Versus Self-Perceived Fertility Problem: Implications of Survey Wording for Assessing Associations with Depressive Symptoms

**DOI:** 10.1089/whr.2020.0032

**Published:** 2020-08-12

**Authors:** Michele H. Lowry, A.L. Greil, J. McQuillan, A. Burch, K.M. Shreffler

**Affiliations:** ^1^ Division of Social Sciences, Alfred University, Alfred, New York, USA.; ^2^ Department of Sociology, University of Nebraska-Lincoln, Lincoln, Nebraska, USA.; ^3^ Division of Counseling and School Psychology, Alfred University, Alfred, New York, USA.; ^4^ Human Development and Family Science, Oklahoma State University, Tulsa, Oklahoma, USA.

**Keywords:** infertility, depressive symptoms, medical criteria, self-perceived fertility problem, regression analysis, National Survey of Fertility Barriers

## Abstract

***Objective:*** To examine how measures of infertility based on medical criteria and based on self-perception relate to depressive symptoms among women with infertility.

***Background:*** Survey-based studies of depressive symptoms have used either measures of self-reported infertility based on meeting medical criteria or measures of self-perceived fertility problems, but seldom both. It is, therefore, not known which type of measure is more closely associated with depressive symptoms.

***Materials and Methods:*** Using ordinary least-squares multiple regression, this study compares associations between a measure of meeting medical criteria for infertility and a measure of self-perceived fertility problems with a common measure of depressive symptoms. Data come from the National Survey of Fertility Barriers, a population-based survey of 4,711 U.S. women.

***Results:*** Both meeting medical criteria for infertility and self-perception were associated with depressive symptoms after controlling for a number of relevant variables, but the coefficient for the self-perception measure was slightly higher than the coefficient for medical criteria.

***Conclusion:*** If possible, both medical criteria and self-perception measures should be used in studies of the consequences of infertility for psychosocial outcomes. If only one measure can be used, self-perception of a fertility problem is an acceptable measure.

## Introduction

Infertility, defined by medical professionals as 12 months of regular, unprotected heterosexual intercourse without conception,^1^ can be a source of psychological distress, particularly for women experiencing involuntary childlessness.^2^ Two types of self-report measures of infertility are available to survey researchers who are interested in the relationship between infertility and depressive symptoms. Some surveys include questions to determine whether a woman meets the medical criteria for infertility.^3^ Others rely on self-reports of perceptions about one's own fertility.^4^ We used data from the National Survey of Fertility Barriers (NSFB), a study of U.S. women that includes both types of measures, to explore how these two different types of measures relate to depressive symptoms.

## Background

Numerous studies have found that women with infertility have higher levels of depressive symptoms than those without it.^5^ Of the 119 women receiving treatment for infertility, 42% had clinically significant levels of distress, as indicated by scores on the General Symptom Inventory.^6^ Women attending an infertility clinic had higher scores for depressive symptoms on the Patient Health Questionnaire than presumably fertile women who attended family practice clinics.^7^ Nulliparous women with infertility, in particular, have reported greater depressive symptoms than those without infertility.^8^

Most studies of infertility and depressive symptoms among women rely on data from clinic-based samples of women under treatment. Clinic-based studies are not representative of all infertile women, however, because fewer than half of infertile women utilize fertility treatment.^9^ To represent all women with infertility in studies of the consequences, it is necessary to use population-based survey data. Because diagnostic measures of infertility are not feasible for women who have not received treatment for survey research, it is necessary to use self-report measures.

One type of self-report measure relies on the medical definition of infertility. Some researchers construct measures of infertility by using contraception and birth histories.^10^ but other researchers simply ask women whether they have experienced a period of regular, unprotected, heterosexual intercourse of at least 12 months.^11^ Other surveys rely on self-reports of perceptions about one's own fertility as distinct from medically defined infertility.^4,10^ Measures based on medical criteria try to come as close as possible to the results that might be obtained through clinical diagnosis,^9^ but these measures do not directly capture the meaning of infertility to participants. Self-perception measures provide the meaning that participants attach to experiences and, as such, reflect individual constructions of fertility. Benyamini^12^ describes perceptions of health status in this way: “These perceptions are not always medically accurate, yet they are rational and internally logical from the person's subjective point of view.” Thus, self-reports of perceived procreative ability provide important information for understanding the psychosocial concomitants of infertility. It is unknown at this point whether measures based on self-perceptions or measures based on medical criteria are more closely associated with depressive symptoms, but it may well be that perceptions of fertility problems matter more for depressive symptoms than meeting medical criteria.

Measures of self-perception do not completely reflect measures based on medical criteria.^13^ In a regional sample, only 35% of women who met medical criteria for infertility perceived themselves to be infertile.^14^ Conversely, women may self-identify as having a fertility problem even if they do not qualify as infertile by medical criteria.^15^ Self-perceptions of fertility problems among young adults are much higher than what prevalence data suggest is the case.^16^ We are not aware of any studies that analyze the relationship between perceived fertility problems and depressive symptoms. This is a serious gap because many surveys measure perceived fertility problems but do not include measures to calculate meeting medical criteria for infertility. It is, thus, important to know whether these measures are associated with depressive symptoms. In this article, we provide the first analysis of measures based on medical criteria and self-perception from the same women and their associations with depressive symptoms.

A number of factors have been shown to be related to depression, responses to infertility, or both. Among women with infertility, higher education and age are associated with lower psychosocial distress.^17,18^ More economic hardship is associated with higher levels of distress.^19^ Religiosity^20,21^ and social support^22,23^ are associated with lower distress for women with infertility. Marriage may make lack of conception more salient, because marriage is often seen as a signal to have children.^24^ Marriage is also associated with better health and less social isolation,^25^ factors associated with lower depressive symptoms. Primary infertility (infertility before having a child) is associated with higher odds of perceiving a problem than secondary infertility (infertility after having a child).^17,26^ In addition, women with primary infertility exhibit higher levels of distress than women with secondary infertility^27,28^ Race/ethnicity is also associated with depressive symptoms.^29,30^

## Materials and Methods

### Sample

The NSFB is based on a random-digit dialing telephone interview of a probability-based sample of 4,711 U.S. women aged 25–45 and focuses on the experiences of fertility and infertility among the women and their partners. There is a second wave with a smaller subset of cases; to have the maximum number of cases we use Wave I, conducted between 2004 and 2007. Interviewing was conducted by the Survey Research Center (SRC) at the Pennsylvania State University and the Bureau of Sociological Research (BOSR) at the University of Nebraska-Lincoln by using the same interviewer training and procedures. Internal Review Boards at both universities approved the study.^*^

The response rate for the screener is 53.7%, which is typical for telephone surveys conducted during the time period.^31^

To assess generalizability, we compared basic demographic characteristics for women ages 25–45 to the comparable age group in the 2005 Current Population Survey (CPS), which uses in-person interviews and has a 90% response rate. There is close correspondence between demographic distributions in both samples. On 22 of 34 demographic characteristics, the difference was within ±1.5%. There was also little difference between the fertility-related variables in the NSFB and similar variables in the National Survey of Family Growth, an in-person interview with a near 90% response rate. Thus, the NSFB sample is similar to well-known nationally representative interview surveys, justifying our confidence in the validity and representativeness of this data set. We used the Dumouchel and Duncan^32^ test to estimate whether or not the variance explained by using the weighted data was significantly different from the variance explained by using unweighted data. The result indicates no difference, so we therefore followed the recommendation of Winship and Radbill^33^ and conducted our analysis by using the unweighted sample.

### Measures

The dependent variable for this study is *depressive symptoms*, measured by using a 10-item modified version of the Center for Epidemiologic Studies–Depression scale (CES-D).^34,35^ The scale included questions such as: “In the past two weeks…I was bothered by things that don't usually bother me;” “I felt depressed;” and “My sleep was restless.” Items were coded or reverse-coded so that high scores indicate high levels of depression. The sum of the items was logged to reduce skew from outliers. Cronbach's alpha for the CES-D scale in the NSFB is 0.78.

The two focal independent variables are (1) *meeting medical criteria for infertility* and (2) *self-perception as having a fertility problem*. Meeting medical criteria entailed answering “yes” to either of the following questions: “Was there ever time when you were trying to get pregnant but did not conceive within 12 months?” and “Was there ever a time when you regularly had sex without birth control for a year or more without getting pregnant?” When asked, “How long did you have sex without using birth control before you got pregnant?” women who responded that it took 12 or more months to get pregnant were also classified as meeting medical criteria. Women who were trying not to become pregnant, who identified themselves as lesbians, or who had been sterilized were not counted as infertile. The analyses excluded 39 women who reported breastfeeding during an episode of infertility from the “meeting medical criteria” category, because breastfeeding can make it much more difficult to conceive.^36^ Breastfeeding, however, does not make it totally impossible to conceive. Therefore, some of the 39 women excluded could actually meet medical criteria for infertility. We ran a sensitivity analysis with these 39 cases included (not shown here) and found that the results of our analysis were substantively the same.

*Perceived fertility problem* was indicated by answering “yes” or “maybe” to either or both of the following questions: “Do you think of yourself as someone who has, has had, or might have trouble getting pregnant?” or “Do you think of yourself as someone who has or has had fertility problems?” Answering “no” to both questions indicated no self-perception of a fertility problem. The “maybe” answer was not provided in the interview but was volunteered by some respondents. Because infertility is a stigmatized condition, we reasoned that women giving “maybe” answers were likely expressing the self-identification of a fertility problem but felt uncomfortable saying “yes.” We, therefore, included “maybe” answers in the “yes” category. We ran a sensitivity analysis in which “maybe” answers were excluded from the “perceived fertility problem” category (not shown here) and found that the results of our analysis were substantively the same.

It is possible that an apparent association between infertility and depressive symptoms is spurious. We, therefore, included as controls a number of variables that have been shown to be related to depressive symptoms. *Age* was measured in years. We treated age as a continuous variable, because grouping ages into categories results in a loss of information. Because the relationship between age and health outcomes is often nonlinear, we ran a sensitivity analysis, including a squared term for age to examine whether the squared term made a difference. It did not make a difference and was, therefore, not included in the analysis. *Education* was measured in years and treated as a continuous variable, because it displayed an approximately normal distribution. Responses to three questions were combined to measure *economic hardship*: (1) “During the last 12 months, how often did it happen that you had trouble paying the bills,” (2) “During the last 12 months, how often did it happen that you did not have enough money to buy food, clothes, or other things your household needed?” and (3) “During the last 12 months, how often did it happen that you did not have enough money to pay for medical care?” Possible answers ranged from 1 (never) to 4 (quite often). This is an additive unidimensional scale with high reliability (*α* = 0.82). Scores range from 3 to 12, with higher scores indicating greater economic hardship.

The *religiosity* scale was developed by the creators of the NSFB and includes four items: “How often do you attend religious services?” “How often do you pray?” “How close do you feel to God most of the time?” and “In general, how much would you say your religious beliefs influence your daily life?” Items were coded so that greater values indicate greater religiosity. Because different items used different scales, items were standardized, and a scale was constructed by using the mean of the standardized items. This measure has an alpha reliability of 0.77. Perceived *social support*, based on Sherburne and Stewart,^37^ was measured by how often the following four kinds of support were available if needed: “someone to give you advice about a crisis,” “someone to give you information to help you understand a situation,” “someone whose advice you really want,” and “someone to share your most private worries and fears with.” Responses range from 1 (often) to 4 (never). Items were reversed-coded so that higher scores indicate greater social support. This is an additive scale that ranges from 4 to 16 (*α* = 0.84).

*Never married* is an indicator variable comparing never married with all other marital statuses. For this and all other indicator variables, a score of “1” indicates that the condition was present, and a score of “0” indicates that is was absent. We did not include separate indicators for divorced and currently married, because these variables refer to the time of the interview rather than the time of the infertility episode. Parity plus 1 is an indicator variable comparing women with any biological children with women without biological children. *Private health insurance* was assessed by the question, “Are you covered by private health insurance, by public health insurance such as Medicaid, or some other kind of health care plan or by no health insurance?” *Employed full-time* was measured by a single binary variable indicating either full-time or part-time employment compared with no employment.

Race/ethnicity was measured by using the two questions used in the 2000 Census.^38^ Individuals who reported multiple races/ethnicities were classified giving first priority to identification as “Hispanic” and second priority to identification as “Black.” Based on this coding, indicator variables were constructed for *Black, Hispanic,* and *Asian* compared with *white*. Those indicating “other” were placed in the “white” category as previous research by using this data set, and this has shown that these two categories are quite similar.^39^ We ran a sensitivity analysis (not shown here) in which we added four additional control variables (family income, want a[nother] child, importance of parenthood, and importance of career). None of these variables was significantly related to depressive symptoms, and the results of regression analyses that included them were substantively the same, so we left them out.

### Plan of analysis

Data were analyzed by using Stata 16. First, we examined the overlap between meeting medical criteria and self-perception *via* a simple cross-tabulation. Next, we described the sample by showing the means and proportions for all variables and by comparing means or proportions for the variables by the four possible infertility groups: neither perceived a problem nor met medical criteria, perceived a problem only, met medical criteria only, and both perceived a problem and met medical criteria. Chi-square and Bonferroni *post hoc* tests indicate similarities and differences among the groups. We did not conduct bivariate analyses of the associations between each control variable and depressive symptoms, because this was not our primary focus. Finally, we examined whether meeting medical criteria or self-perception of a fertility problem has a stronger association with depressive symptoms by using a series of ordinary least squares (OLS) multiple regression models. Using a series of OLS models provides a way to assess whether the measures of infertility add to models with conventional correlates of depressive symptoms, whether the measures of infertility have independent or combined associations with depressive symptoms, and whether the focal associations are mediated or explained by the control variables, and are appropriate for a continuous dependent variable. We ran the following models: (1) control variables only, (2) medical criteria only, (3) self-identification only, (4) medical criteria and self-identification, and (5) medical criteria, self-identification, plus an interaction term for medical criteria*self-identification.

## Results

Table 1 displays the various combinations of meeting medical criteria for infertility and perceiving a fertility problem. Most of the women neither met medical criteria for infertility nor saw themselves as having a fertility problem (42%). Similar percentages of women met criteria with (24%) and without (27%) perceiving a problem. A few women perceived a problem without meeting medical criteria (8%).

**Table 1.** Cross-Tabulation of Self-Identfying as Having a Fertility Problem and Meeting Medical Criteria for Infertility: 4,711 Women, National Survey of Fertility Barriers

Percieves a fertility problem	Meets medical criteria		
	Yes	No	Total
Yes			
*n*	1,996	1,253	3,249
%	42	27	69
No			
*n*	354	1,108	1,462
%	8	24	31
Total			
*n*	2,359	1,261	4,471
%	50	59	100

Table 2 displays descriptive statistics for the total sample and for the four possible combinations of meeting medical criteria for infertility and perceiving a fertility problem. We show means and standard deviations for continuous variables and proportions for categorical variables. The mean score for CESD-10 among the sample as a whole was 7.06, comparable to the mean found in another study of CES-D among infertile women.^40^ Among the sample, 27% of the women met the clinical threshold for depressive systems. This is consistent with other studies that reported CESD for infertile women.^41,42^ The mean age of the women in the sample was about 36; the average educational attainment was a little less than 3 years of college. About 79% of the sample had ever been married, and almost three quarters had at least one child. Almost three quarters had private health insurance, and 58% were employed full-time. Women who identified as white made up 57% of the sample, 20% identified as Black, 18% as Hispanic, and 2% as Asian.

**Table 2.** Means and Proportions for Variables in the Analyses by Self-Perceiving Fertility Problems and/or Meeting Medical Criteria for Infertility

	Neither self nor medical		Self-perceives a fertility problem		Meets medical criteria for infertility		Self-perceives and meets medical criteria		Total	
	Mean	SD	Mean	SD	Mean	SD	Mean	SD	Mean	SD
Continuous variables										
Depressive symptoms (0–30)	6.28	4.54	7.32	5.05	7.53_a_	0.27	8.07_a_	5.8	7.06	5.07
Age (25–45)	34.46_a_	6.08	35.61_b_	5.9	34.96_ab_	6.12	35.54	5.6	35.29	5.98
Education (years: 0–22)	15.11_a_	2.84	13.88	2.8	15.32_a_	3.14	14.45	2.73	14.62	2.8
Economic hardship (3–12)	4.43	1.61	5.03_a_	1.86	4.75_a_	1.8	4.98a	1.98	4.74	1.81
Religiosity (−2.37 to 1.08)	−0.07_a_	0.72	0.12	0.6	−0.12_a_	0.74	0.03	0.68	0	0.07
Social support (4–16)	14.67_a_	2.02	14.22_b_	2.47_a_	14.6	2.06	14.18_b_	2.54	14.43	2.29
Categoricl variables										
Met clinical threshhold for CESD-10	0.21		0.30_a_		0.32_a_		0.33_a_		0.27	
Never married	0.24_a_		0.21_a_		0.33		0.13		0.21	
Parity 1+	0.69		0.9		0.48		0.73		0.74	
Private insurance	0.79_a_		0.66		0.81_a_		0.72		0.74	
Employed full-time	0.60_a_		0.55_b_		0.63_ac_		0.55_bc_		0.58	
Black	0.16_a_		0.28		0.15_a_		0.20		0.20	
Hispanic	0.17_a_		0.22		0.16_a_		0.16_a_		0.18	
Asian	0.03_a_		0.02_a_		0.02_a_		0.03_a_		0.02	
White	0.62_ab_		0.45_a_		0.65_b_		0.59		0.57	
*n*	1,996		1,253		354		1,108			

Data source: National Survey of Fertility Barriers, cases with complete data, *n* = 4,711.

For continuous data, means that do not share a subscript are significantly different from each other by using the Bonferroni *post hoc* test. Proportions that do not share a subscript are significantly different from each other by using chi-square.

CES-D, Center for Epidemiologic Studies—Depression scale; SD, standard deviation.

Women who perceived a fertility problem, who met medical criteria for infertility, or both, perceived a problem, and met medical criteria exhibited more depressive symptoms than women who neither perceived a problem nor met medical criteria. Women who both met medical criteria for infertility and perceived a fertility problem had the highest level of depressive symptoms. Those who self-perceived only had fewer symptoms than those who both self-perceived and met medical criteria. Women who self-perceived had similar levels of depressive symptoms compared with those meeting medical criteria, and those who met medical criteria had similar levels of depressive symptoms compared with those who both self-perceived and met medical criteria. The proportion of women at or above the clinical threshold was lower in the “neither” category than in the other three groups.

The patterns of differences among the four fertility status groups varied from variable to variable, and space does not permit detailed discussion here. There were significant variations by fertility status group for all variables in the analysis except one: proportion Asian. That variation was found from group to group suggests that it is, indeed, important to control for these variables in the regression models.

Table 3 presents the results of OLS multiple regression analyses. In Model 1, several of the control variables were associated with depressive symptoms. Greater economic hardship, never having been married, and having at least one child were associated with more depressive symptoms, but higher education, religiosity, social support, having private insurance, being employed full-time, and claiming Hispanic or Asian ethnicity were associated with fewer depressive symptoms. Models 2 (meeting medical criteria for infertility) and 3 (self-identifying as having a fertility problem) show that each measure of infertility was associated with higher depressive symptoms. The associations for the control variables remained similar for both models, but for Model 2, having a child was now associated with higher depressive symptoms and claiming Hispanic ethnicity was no longer associated with depressive symptoms. For Model 3, claiming Hispanic or Asian ethnicity was no longer associated with depressive symptoms.

**Table 3.** Logged Depressive Symptoms by Medical Infertility, Self-Identified Fertility Problem, and Control Variables

	Model 1				Model 2				Model 3				Model 4				Model 5			
	B	SE	β		B	SE	β		B	SE	β		B	SE	β		B	SE	β	
	Controls only				Controls and medical criteria				Controls and selfID				Controls, medical, and selfID				Controls, medical, selfID and medical × selfID			
Medical criteria for infertility^a^					0.10	0.02	0.06	^***^					0.06	0.02	0.04	^*^	0.07	0.03	0.04	^*^
Self-identified a fertility problem^b^									0.14	0.02	0.08	^***^	0.12	0.03	0.07	^***^	0.15	0.04	0.09	^**^
Medical criteria × selfID																	−0.05	0.05	−0.03	
Age	0.00	0.00	0.00		0.00	0.00	0.00		0.00	0.00	0.00		0.00	0.00	−0.01		0.00	0.00	−0.01	
Education	−0.02	0.00	−0.07	^***^	−0.02	0.00	−0.07	^***^	−0.02	0.00	−0.07	^***^	−0.02	0.00	−0.07	^***^	−0.02	0.00	−0.07	^***^
Economic hardship	0.09	0.01	0.20	^***^	0.09	0.01	0.20	^***^	0.09	0.01	0.19	^***^	0.09	0.01	0.19	^***^	0.09	0.01	0.19	^***^
Religiosity	−0.11	0.02	−0.10	^***^	−0.12	0.02	−0.10	^***^	−0.12	0.02	−0.10	^***^	−0.12	0.02	−0.10	^***^	−0.12	0.02	−0.10	^***^
Social support	−0.03	0.01	−0.09	^***^	−0.03	0.01	−0.09	^***^	−0.03	0.01	−0.08	^***^	−0.03	0.01	−0.08	^***^	−0.03	0.01	−0.08	^***^
Never married	0.09	0.03	0.04	^***^	0.10	0.03	0.05	^**^	0.10	0.03	0.05	^**^	0.11	0.03	0.05	^***^	0.10	0.03	0.05	^**^
Parity 1+	0.06	0.03	0.03	^*^	0.05	0.03	0.03		0.08	0.03	0.05	^**^	0.07	0.03	0.04	^**^	0.07	0.03	0.04	^*^
Private insurance	−0.10	0.03	−0.05	^**^	−0.09	0.03	−0.05	^**^	−0.10	0.03	−0.05	^**^	−0.10	0.03	−0.05	^*^	−0.10	0.03	−0.05	^**^
Employed full-time	−0.08	0.02	−0.05	^**^	−0.08	0.02	−0.05	^**^	−0.08	0.02	−0.05	^**^	−0.08	0.02	−0.05	^*^	−0.08	0.02	−0.05	^**^
Black^c^	0.00	0.03	0.00		−0.01	0.03	−0.01		0.00	0.03	0.00		0.00	0.03	0.00		0.00	0.03	0.00	
Hispanic^c^	−0.06	0.03	−0.03	^*^	−0.07	0.03	−0.03	^*^	−0.06	0.03	−0.03		−0.06	0.03	−0.03		−0.06	0.03	−0.03	^*^
Asian^c^	−0.04	0.08	−0.01	^***^	−0.05	0.08	−0.01		−0.05	0.08	−0.01		−0.05	0.08	−0.01		−0.05	0.08	−0.01	^***^
Constant	2.05	0.13	.	^***^	2.01	0.13	.	^***^	2.01	0.13		^***^	1.99	0.13		^***^	1.99	0.13	.	^***^
*R*-square	0.10				0.10				0.11				0.11				0.11			

Dependent variable: log of depressive symptoms (CESD-10).

^a^ Reference category is not meeting medical criteria for infertility.

^b^ Reference category is not self-identifying a fertility problem.

^c^ Reference category is White or other race/ethnicity.

^*^ *p* < 0.05; ^**^p < 0.01; ^***^p < 0.001.

*B*, unstandardized regression coefficient; *β*, standardized regression coefficient; SE, standard error.

Model 4 shows that when both measures of infertility are in the model together, they each have significant associations with depressive symptoms (*p* < 0.05). A Wald test showed that the coefficient of the association between self-perception and depressive symptoms was significantly larger than the association between meeting medical criteria and depressive symptoms [*F*(2, 1,469) = 19.22; *p* = 0.000]. The only change in the control variables between Model 1 and Model 4 was that the associations between claiming Hispanic or Asian ethnicity and depressive symptoms were no longer significant. The combined effect of meeting medical criteria and perceiving a fertility problem (the interaction) was not statistically significant (see Model 5). Therefore, there is no added consequence from meeting medical criteria and perceiving a fertility problem simultaneously. However, adding the interaction term did result in the measures of Hispanic or Asian ethnicity and depressive symptoms being significant in Model 5.

## Discussion

Multiple regression analysis, controlling for variables associated with infertility and depressive symptoms in prior research, revealed that both meeting medical criteria and perceiving a fertility problem were associated with higher depressive symptoms. The association between self-perception and depressive symptoms was significantly larger than the association between meeting medical criteria and depressive symptoms. There was no additional contribution from the interaction of the two measures.

The analyses indicate that perceiving a problem, which captures women's subjective experience of fertility problems, is associated with depressive symptoms even after controlling for whether or not a woman responds to questions in a way that indicates meeting medical criteria for infertility. We also find that, when self-perception and meeting medical criteria are included in the same model, self-perception accounts for more of the variation in depressive symptoms than meeting medical criteria. Therefore, for women, perceptions of fecundity seem to be more consequential for psychological distress than fecundity itself. Confidence in this finding will increase with analyses using multiple samples and measures. In the meantime, it is useful to know that self-perception measures of infertility can be useful for studies of the psychological consequences of infertility meanings and experiences. Because both self-perception and meeting medical criteria are associated with depressive symptoms, researchers investigating infertility and depressive symptoms would be well advised to use both types of measures. If only one measure can be used because of cost or time constraints, the results suggest that perception of a fertility problem captures more of the experience of infertility, especially when the primary goal of the research is to assess psychosocial experiences of infertility.

We were somewhat surprised to find no interaction between self-perception and meeting medical criteria. One might expect that meeting medical criteria would be more strongly related to depressive symptoms among women who also perceived a problem. It is not clear why this was not the case. It is possible that our results are biased by the omission of control variables associated with both the dependent and independent variables. We tried to minimize this problem by running sensitivity analyses in which possible confounders were added to the model. Adding these potential confounders did not substantively change the associations, but the possibility of undetected endogeneity still remains. Further research is required to clarify this issue.

The utility of our study is limited by having only a single cohort of women of childbearing years. The data on which this study is based are now about 15 years old, and the social context of infertility may have changed enough to affect the meaning of infertility and its relationship to depressive symptoms. Cross-sectional data prevent strong conclusions about causal associations, but the measure of depressive symptoms captures the “last two weeks” and the measures of perceived and medical criteria infertility refer to any time in the past or present. In addition, central concepts were sometimes measured at the time of the infertility episode and sometimes at the time of the interview. Thus, some apparent associations might be misleading. Even though we ran sensitivity analyses and found that various methods of classifying respondents as infertile and placing them into one of the four groups did not change conclusions, it is still possible that some cases were misclassified. It must also be noted that many of our effect sizes were relatively small.

This analysis is also limited by the fact that we had only one measure of depressive symptoms available to us. It would strengthen our argument about the relative sensitivity of our two measures of infertility if we were to find similar patterns of association using other measures of depressive symptoms. In the absence of a “gold standard” for measuring infertility, we cannot make a strong claim that one measure is superior to the other. We must limit ourselves to the claim that depressive symptoms, as measured by the CESD-10, are more sensitive to differences in perceived fertility problems than they are to differences in a measure of medical criteria. Still, this study contributes the first systematic analysis of the relative strength of the association between two measures of infertility and psychological distress. Our finding that self-perceived fertility problems are more strongly associated with depressive symptoms than meeting medical criteria suggests the importance of taking self-perceptions into account when examining the psychological concomitants of infertility. In addition, if a study only has access to one but not the other measure of infertility, it is still possible to assess implications for depressive symptoms.

## Abbreviations Used

B: unstandardized regression coefficient
β: standardized regression coefficient
BOSR: Bureau of Sociological Research
CES-D: Center for Epidemiologic Studies—Depression scale
CPS: Current Population Survey
NSFB: National Survey of Fertility Barriers
OLS: ordinary least squares
SD: standard deviation
SE: standard error
SRC: Survey Research Center

## Notes

^*^ The data are publicly available at: https://www.icpsr.umich.edu/icpsrweb/DSDR/studies/36902#bibcite.
